# Carbon Nanotubes/Regenerated Silk Composite as a Three-Dimensional Printable Bio-Adhesive Ink with Self-Powering Properties

**DOI:** 10.1021/acsami.1c03288

**Published:** 2021-05-03

**Authors:** Silvia Bittolo Bon, Irene Chiesa, Micaela Degli Esposti, Davide Morselli, Paola Fabbri, Carmelo De Maria, Antonino Morabito, Riccardo Coletta, Martino Calamai, Francesco Saverio Pavone, Rodolfo Tonin, Amelia Morrone, Giacomo Giorgi, Luca Valentini

**Affiliations:** † Dipartimento di Ingegneria Civile e Ambientale, Università degli Studi di Perugia, Strada di Pentima 4, Terni 05100, Italy; ‡ Italian Consortium for Science and Technology of Materials (INSTM), Via Giusti 9, Firenze 50121, Italy; § Department of Ingegneria dell’Informazione and Research Center E. Piaggio, University of Pisa, Largo Lucio Lazzarino 1, Pisa 56122, Italy; ∥ Department of Civil Chemical, Environmental and Materials Engineering (DICAM), Università; di Bologna, Via Terracini 28, Bologna 40131, Italy; ⊥ Department of Pediatric Surgery, Meyer Children’s Hospital, Viale Pieraccini 24, Firenze 50139, Italy; # Dipartimento Neuroscienze, Psicologia, Area del Farmaco e della Salute del Bambino NEUROFARBA, Università degli Studi di Firenze, Viale Pieraccini 6, Firenze 50121, Italy; ¶ School of Health and Society, University of Salford, Salford M5 4WT, United Kingdom; ∇ European Laboratory for Non-linear Spectroscopy (LENS), University of Florence, Sesto Fiorentino (FI) 50129, Italy; ○ National Institute of Optics -National Research Council (CNR-INO), Sesto Fiorentino (FI) 50129, Italy; ⧫ Department of Physics, University of Florence, Sesto Fiorentino (FI) 50121, Italy; †† Molecular and Cell Biology Laboratory, Paediatric Neurology Unit and Laboratories, Neuroscience Department, Meyer Children’s Hospital, Firenze 50139, Italy; ‡‡ Dipartimento di Ingegneria Civile e Ambientale (DICA), Università degli Studi di Perugia, Via G. Duranti 93, Perugia 06125, Italy; §§ CNR-SCITEC, Perugia I-06123, Italy

**Keywords:** regenerated silk, carbon nanotubes, mechanical properties, 3D printing, interface modeling, self-powering bio-adhesives

## Abstract

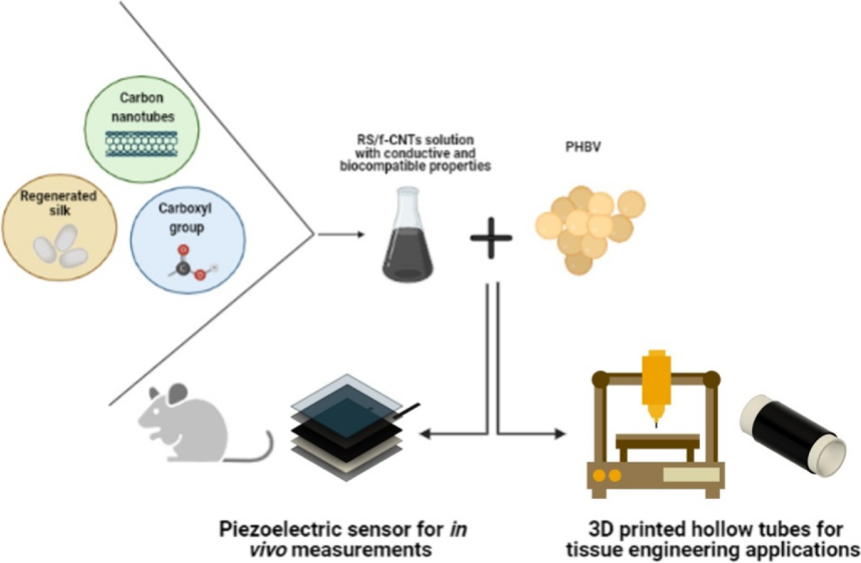

In this study, regenerated silk (RS) obtained from *Bombyx Mori* cocoons is compounded with carboxyl-functionalized carbon nanotubes (f-CNTs) in an aqueous environment for the fabrication of functional bio-adhesives. Molecular interactions between RS and carboxyl groups of CNTs result in structural increase of the β-sheet formation, obtaining a resistant adhesive suitable for a wet biological substrate. Moreover, the functionalization of CNTs promotes their dispersion in RS, thus enabling the production of films with controlled electrical conductivity. The practical utility of such a property is demonstrated through the fabrication of a piezoelectric device implanted in a rat to monitor the breathing in vivo and to be used as a self-powered system. Finally, RS/f-CNTs were used as a printable biomaterial ink to three dimensionally print bilayer hollow tubular structures composed of poly(3-hydroxybutyrate-*co*-3-hydroxyvalerate) (PHBV) and RS. Initial tests carried out by seeding and growing human skin fibroblasts demonstrated that the 3D printed bilayer hollow cylindrical structures offer a suitable surface for the seeded cells to attach and proliferate. In general, the herein proposed RS/f-CNT composite serves as a versatile material for solvent-free dispersion processing and 3D printing, thus paving a new approach to prepare multifunctional materials with potential applications of great interest in sealing biological substrates and implantable devices for regenerative medicine.

## 1 Introduction

In the last decade, adhesives have gained increasing interest in many surgical applications.^1−9^ Synthetic adhesives (i.e., Coseal, DuraSeal, and Tisseel), for example, are currently used as sealants in anastomosis, that is a new connection between two body structures that carry fluid.^10,11^ However, the lack of adhesion on a wet surface as well as the elastic modulus mismatch between the substrate and adhesive film still make the development of a suitable material quite a challenging task.^12,52^

In this context, the use of biopolymers or natural polymers can add essential properties to the adhesive such as biocompatibility and biodegradation.^7−9^

A natural-based adhesive that can be sprayed or deposited on an anastomosis would pave the way to suture-less anastomosis, thus facilitating the surgeon’s tasks.^10−12,52^ This natural-based adhesive could also act as a sealant reducing the risk of anastomotic leak, which is still the most feared postoperative complication in intestinal surgery.^13^ In the long run, the realization of a smart adhesive for in situ health monitoring is another major objective. This application will benefit by the design of custom and complex geometries, achievable by advanced three-dimensional (3D) printing technologies.^14^

Silk is a natural and hierarchical material made of fibrils with multilength scales that can serve as a filler once introduced in a homogeneous matrix.^15^ Several research studies focused on the extraction and solubilization methods to obtain fibroin protein from silk that assembled in regenerated silk (RS) and are then used as building blocks in gels, films, or fibers.^16^ Due to its biocompatibility, and reabsorbing properties, fibroin has been widely used in tissue engineering.^17^ However, the fabrication of fibroin protein requires solvents and downstream processes that limit the solubility and the dispersion stability of nanomaterials in the hosting fibroin protein.

Among different nanofillers, carbon nanotubes (CNTs) are a conductive nanomaterial with excellent mechanical properties.^18,19^ In addition, CNTs have been demonstrated to be biocompatible^20,21^ and have already been used as a 3D printing ink in the fabrication of green electronic devices and biosensors.^22−24^ For example, Wei et al. fabricated an integrated strain sensor via extrusion-based 3D printing for in situ monitoring of finger flexion, knee flexion, and respiration by resuspending CNTs into polyacrylic acid and sodium alginate.^25^

However, due to their hydrophobic nature, CNTs are difficult to disperse in aqueous environments if not properly functionalized. Furthermore, nonfunctionalized CNTs have the strong tendency to form aggregates in biological environments, which limit their practical applications in this area.

It is well known that carboxyl-functionalized carbon nanotubes (f-CNTs) can be easily dispersed in water due to the presence of oxygen-containing functional groups that enhance the interactions at the interface with the hosting matrix.^26^

The combination of the aforementioned points allows us to envisage a solvent-free method for the development of biocompatible conductive adhesives for wet tissues.

In this study, we report a water-based method to disperse f-CNTs in a silk fibroin water solution to prepare biocompatible adhesives for wet-substrates, characterized by piezoelectric properties. More in detail, RS was re-dispersed in the water environment to promote the dispersion of f-CNTs to prepare RS/f-CNTs composites. The proposed materials demonstrated to be electrically conductive and showed remarkable biocompatibility, adhesive properties on biological wet substrates, and mechanical properties (i.e., shear strength) comparable with those of their synthetic counterparts, thus promoting their integration in functional devices. To further demonstrate the versatility of the developed compound, RS/f-CNTs have been used as a biomaterial ink for the 3D printing of bilayer hollow cylindrical structures with potential applications in tissue engineering.

## 2 Experimental Section

### 2.1 Material Preparation

Silk cocoons were supplied from a local farm. Sodium hydrogen carbonate (NaHCO_3_), calcium chloride (CaCl_2_), formic acid (FA), chloroform (CHCl_3_), and poly(3-hydroxybutyrate-*co*-3-hydroxyvalerate) (PHBV) of custom-grade were supplied by Sigma-Aldrich. Short COOH-functionalized multiwalled CNTs (outer diameter ≈ 1–4 nm, length 0.5–2 μm, functional content 2.73 wt %) were supplied by Cheap Tubes Inc. Unmodified CNT aqueous ink (CNTs, viscosity 1 mPa·s, electrical conductivity <400 Ω/sq) was supplied by Sigma-Aldrich. PHBV was carefully purified by the procedure described elsewhere.^27^

Before solubilization, silk cocoons were treated with NaHCO_3_ (5 g in 200 mL of water) in boiling water for 30 min and rinsed with deionized water to eliminate the sericin, the nonfibrous component; this procedure was repeated two times. The so-obtained fibers were then left to dry at room temperature.

RS was produced by dispersing the degummed silk fibers into the FA/CaCl_2_ solution by magnetic stirring at room temperature for 5 min to obtain a homogeneous solution; the CaCl_2_ amount was calculated as 25% with respect to the silk amount (0.65 g), and it was dissolved in FA (5 mL). The solution was cast onto a polystyrene dish (5 cm diameter) at room temperature, and the FA solution was left to evaporate for 8–12 h. Afterward, the dried films were heated at 60 °C for 2 h to remove the possible residual solvent. Films with a thickness of approx. 100 μm were obtained. RS was then re-dispersed in water (2 mL) and 1 wt % (calculated with respect to the silk) of f-CNTs was added. RS/f-CNTs were bath-sonicated for 30 min at room temperature with a frequency of 60 Hz. For comparison purposes, CNTs were added to RS water dispersion. Hereinafter, we refer to RS/CNTs and RS/f-CNTs for samples obtained by compounding RS with unmodified and −COOH modified CNTs, respectively.

### 2.2 Material Characterization

The RS and RS composite films were characterized using a Fourier transform infrared spectrophotometer (JASCO FT/IR 615, Oklahoma City, OK, USA) in attenuated total reflection mode: the collected spectra were analyzed in the range 1750 to 1520 cm^–1^ (amide I and amide II bands). The spectra were deconvoluted by first smoothing the signal with a polynomial function with a 15-point Savitski–Golay smoothing function, subtracting a linear baseline, and applying a Gaussian deconvoluting curves by Origin 9 software.

The mechanical properties of the undoped and composite RS films were measured with a tensile testing machine (Lloyd Instr. LR30K, UK). Rectangular samples (1.5 cm × 3 cm × 100 μm) were stretched with a strain rate of 5 mm·min^–1^ using a 500 N load cell. Four samples per composition were tested.

The morphology of the prepared films was investigated by a high-resolution scanning electron microscope equipped with a cold field emission gun (Supra 35), applying an accelerating voltage of 5 kV. The electrical properties of RS, RS/CNTs, and RS/f-CNTs films were tested by a Keithley 6517 Hi-R test on circular shaped samples.

The adhesive properties were tested by gluing two portions of the porcine intestine with neat and composite RS gels. Once the materials have been applied between porcine intestines, the structures were stored in a climatic chamber for 24 h at 37 °C and relative humidity of 65%. The shear strength was then calculated by a tensile test (with a strain rate of 5 mm·min^–1^), dividing the maximum force by the adhesion area. To avoid damages of the tissues by the clamping, polystyrene stiff films were glued to the back of the intestine as the substrate. The accurate values of the adhesion areas were evaluated by importing the digital photos in AutoCAD (Autodesk Inc.). The results are the average of at least four measurements per composition of adhesive.

Thermal properties of neat and composite RS films were evaluated by differential scanning calorimetry (DSC, Q10, TA Instruments) equipped with a Discovery Refrigerated Cooling System (RCS90, TA Instruments). Samples of approx. 15 mg were placed into aluminum pans and subjected to a heating cycle from −10 °C to +150 °C (hold for 1 min) with a heating rate of 5 °C·min^–1^. The DSC cell was purged with dry nitrogen at 50 mL·min^–1^. Before the measurements, the system was calibrated both in temperature and enthalpy with indium standard. DSC curves were processed with TA Universal Analysis 2000 software (TA Instruments) in order to extrapolate the glass transition temperature (*T*_g_) from the typical enthalpic jump associated to the transition from the glassy state to the rubbery state. *T*_g_ was determined as the mean value between the onset point and endpoint of the glass transition signal.

In order to investigate the possibility to sterilize the neat and composite RS by using germicidal radiations, samples were irradiated in air with ultraviolet light (UV-C). UV treatment was performed using an UV-C lamp (SANKIO DENKI G15T8 lamp, Japan, ultraviolet output 4.9 W, mounted into a cabinet model SafeFAST Classic 212, DASITgroup, Italy) with an irradiation wavelength centered at 253.7 nm. Films were placed at 60 cm apart from the UV source and irradiated on both sides for 20 min each. DSC investigations were also performed on UV-irradiated specimens by using the procedure previously described.

### 2.3 Fabrication and Characterization of Piezoelectric Devices

The piezoelectric device was fabricated as already described elsewhere.^28^ Briefly, films of PHBV with a thickness of approx. 300 μm were prepared by solvent-casting^28^ and were used as a substrate. Neat and composite RS solutions were then drop-cast on the PHBV films and left to evaporate at 40 °C for 12 h. Carbon tapes were attached on the top and bottom side of the bilayer films. After that, Cu wires were attached to the bottom and top sides of the carbon tape using silver paste.

Animal experiments were approved by the Italian Ministry of Health (Prot. Numb. n° 226/2020-PR) and were performed in accordance with the EU Directive 2010/63/EU for animal experiments guidelines. To respect the ethical imperative to use the minimum necessary number of animals, power calculation analysis was performed to predict the adequate experimental power and sample number. Albino-Wistar rats (average weight 320 g) were considered for this experiment. All the animals had free access to food and water. All surgical procedures were performed under general anesthesia with inhaled oxygen and 5% isoflurane. Respiratory support during the experiments was performed by a facial mask using an oxygen flow of 1–2 ml·min^–1^ and 1–3% isoflurane.

A 2 × 2 cm^2^ subcutaneous pocket was performed in the lateral side of the rat abdomen. The piezoelectric device was gently slidden into the pocket, allowing for the terminal end to be connected to the Keithley 4200 semiconductor characterization system. The so-obtained piezoelectric devices were tested by measuring the open-circuit voltage signal between the two Cu wires by a Keithley 4200 Semiconductor Characterization System.

### 2.4 Computational Details

Geometry optimization of the interfaces and of the interface components (i.e., nanotube and fibroin) was performed by means of density functional theory-based simulations as implemented in the Vienna Ab-initio Simulation Package (VASP) code.^29−32^ The projector augmented wave (PAW) method^33^ along with the generalized gradient approximation exchange–correlation functional as parametrized by Perdew–Burke–Ernzerhof (PBE)^34^ plus the DFT-D3 dispersion correction to include the van der Waals interactions^35,36^ was employed. A plane-wave cutoff energy of 600 eV was similarly used in the calculations. The interfaces were assembled as follows. All the structures were optimized until the forces on all atoms were smaller than 0.04 eV/Å.

#### 2.4.1 Carbon Nanotube

We assembled and optimized a (9,9) chirality nanotube^37^ and constructed, by re-optimizing it, the 1 × 1 × 2 supercell. The diameter of the tube is ∼12.2 Å. The periodic *c* vector is 7.38 Å, while sufficient vacuum was added on top of the other two nonperiodic directions in order to avoid any possible spurious interaction with the tube replicas.

#### 2.4.2 Silk Fibroin

To model the silk fibroin, we considered the structure previously reported by Asakura et al.^38^ In detail, we optimized the unit cell of the fibroin (*Z* = 4), for which we obtained the following lattice parameters; *a* = 9.19 Å, *b* = 8.68 Å, and *c* = 7.01 Å, respectively.^38^ Then, we assembled a bilayer adding vacuum still along the nonperiodic, perpendicular to the surface, direction.

#### 2.4.3 CNT/Silk Fibroin Interface

To assemble the final interface, we considered a 4 × 1 supercell of the fibroin bilayer (4 × *a* = 36.77 Å) applying the previously optimized nanotube *c* parameter (7.38 Å) to the fibroin. Due to the one-dimensional periodicity of the tube, the overall (tensile) mechanical stress at the interface is only ascribed to such mismatch (∼5% due to the physisorbed nature of the interface, no chemical stress may be taken into account in calculations) that corresponds to ∼1.5 × 10^–3^ eV/atom. According to the very large interface area, the so-assembled systems were optimized sampling the Brillouin zone only with the Γ-point. We properly corrected the eventual residual dipole present at the interfaces along the nonperiodic direction.

Carboxyl-functionalized tubes were considered with a COOH concentration that reflects the experimental data.

A key parameter to evaluate the stability of the interface is the so-called adhesion energy (*E*_adh_). The adhesion energy of the interface, that is, the opposite of the energy required to separate the surfaces that form the interface, is calculated according to the formula
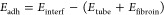
1where *E*_interf_ is the energy of the optimized interface, while *E*_tube_ and *E*_fibroin_ are the energies of the C nanotube and of the fibroin bilayer, respectively.^39^ A last feature we calculated is the Bader charge^40,41^ distribution that we evaluated on both interface systems and separated constituents. To do it, we increased 40% of the standard grid points in the “fine” fast Fourier transform grid along the lattice vectors.

### 2.5 3D Printing and Characterization of PHBV and RS/f-CNTs

Hollow bilayer cylindrical structures were 3D-printed using a piston-driven extrusion-based 3D printer, featuring a rotating spindle (spindle diameter = 5 mm, spindle speed = 15 rpm, nozzle diameter = 0.8 mm). The inner layer was obtained by extruding a 150 mg·mL^–1^ solution of PHBV in chloroform, whereas the external layer was made of a RS/f-CNT-based ink. PHBV tubular monolayer structures were fabricated as control.

In order to evaluate the biocompatibility of the tubes, samples were sterilized by 1 h of exposure to the UV-C in a Biosafety Level 2 cabinet. Anonymous human skin fibroblast cell lines (henceforth referred to as fibroblasts) were used in compliance with the Ethical recommendations issued and the International Declaration on Human Genetics Data of 2003. Fibroblasts were seeded and grown on both surfaces of each sample under culture conditions with Dulbecco’s modified Eagles (DMEM) with fetal bovine serum (10%) and antibiotics for 21 days in a 12-well plate. DMEM media were changed every 3–4 days. Fibroblast growth and confluence were evaluated daily by an inverted microscope. Samples incubated with cells were washed with phosphate-buffered saline (PBS) and fixed using 4% paraformaldehyde. After rinsing with PBS and blocking with a 4% solution of bovine serum albumin in PBS, the samples were fluorescently labeled after simultaneous incubation for 15 min with Hoechst 33342 (Thermofisher) at a concentration of 1 μg·ml^–1^, and with Wheat Germ Agglutinin 568 (WGA568, Thermofisher), at 5 μg·ml^–1^. Imaging was performed with a confocal microscope (Nikon Eclipse TE300), equipped with the Nikon C2 scanning head Coherent CUBE (diode 405 nm) and Coherent Sapphire (Sapphire 561 nm) lasers. Emission filters for imaging were 452/45 nm and 595/60 nm. In order to test the biodegradation of the material, the RS and RS composite films were kept in PBS, 1× Solution, pH 7.4, and, using a six-well plate, incubated at 37 °C. Mass was measured daily with an analytical balance. Before being weighed, the samples were dried with a blotting paper.^42,43^

## 3 Results and Discussion

### 3.1 RS Composites

**Figure 1:**
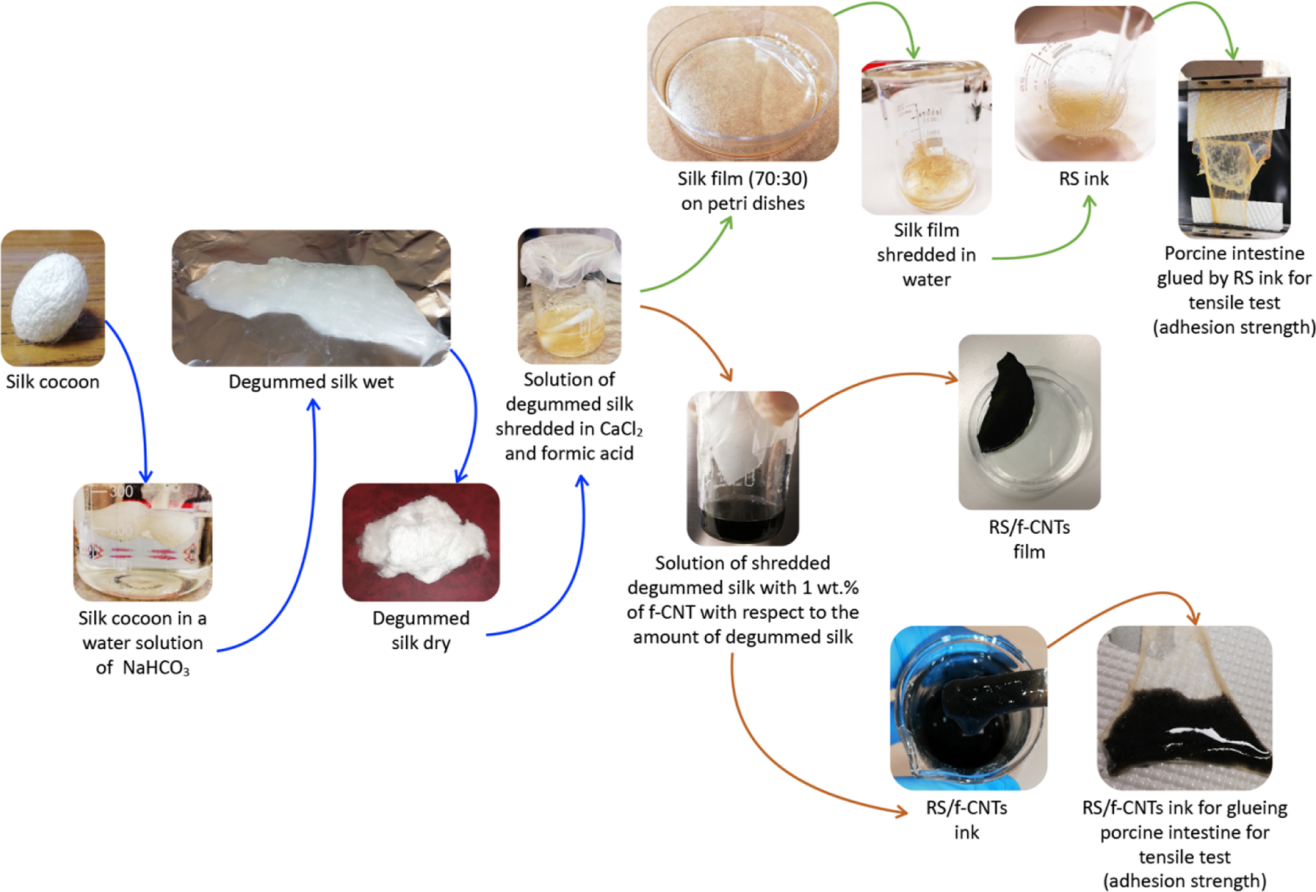
Schematic illustration of the solvent-free fabrication method of RS/f-CNT dispersion. Silk cocoons were degummed and solubilized in FA and left to evaporate. RS was then re-dispersed in water by adding f-CNTs followed by sonication; the RS/f-CNTs can take different consistencies from ink to thin solid films characterized by high flexibility.

In Figure 1, we report a schematic illustration of the water-based method that we developed to produce RS composite gels. Briefly, dried degummed silk was used to obtain a stable solution of RS that was employed to prepare RS films. Consequently, the RS film was re-dispersed in water to obtain a gel with adhesive properties. Unfunctionalized CNTs and f-CNTs were then compounded with RS in a water environment to get a stable black dispersion that was also used as an adhesive.

**Figure 2:**
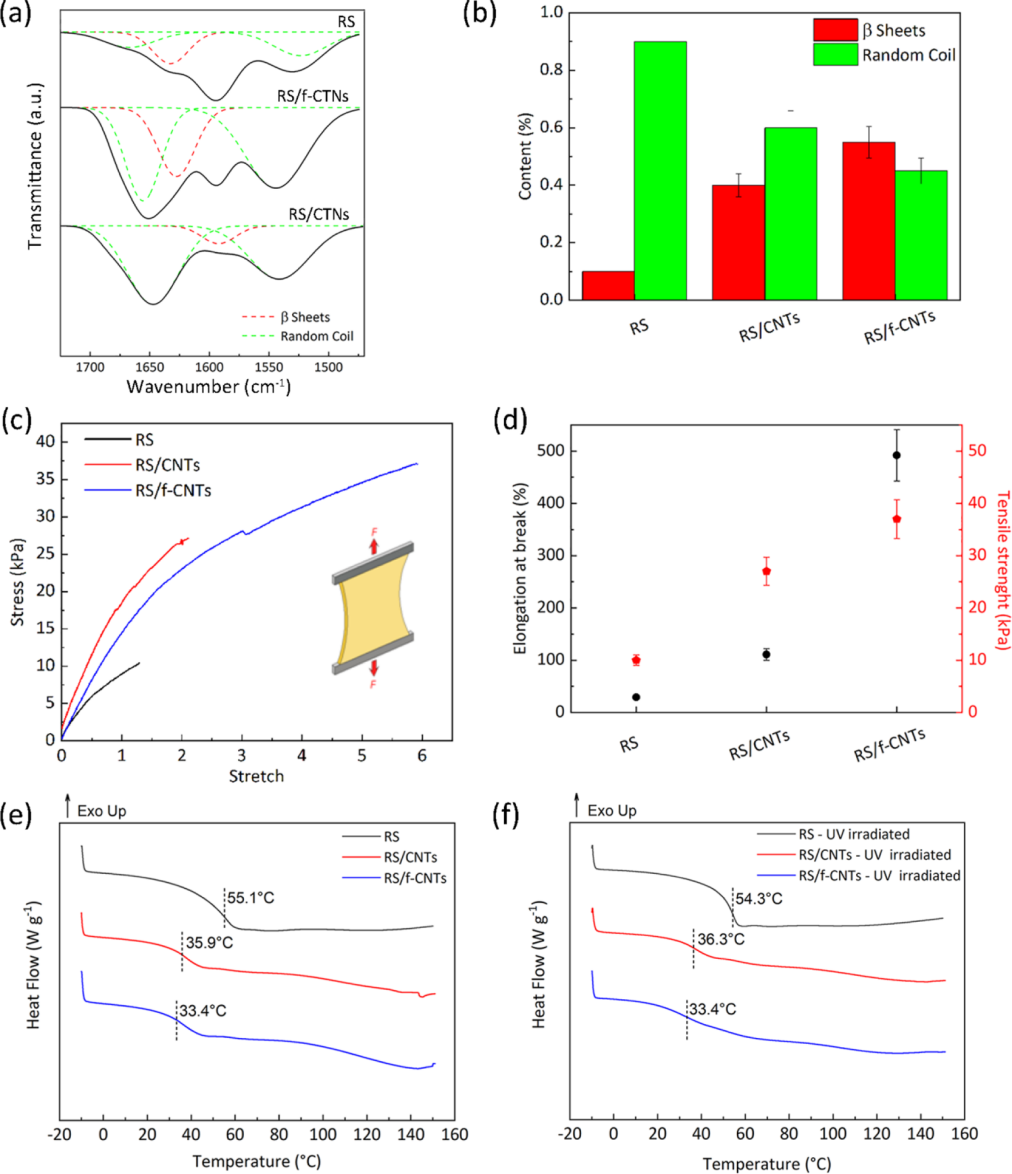
(a) FTIR spectra in amide-I and amide II regions of RS, RS/f-CNTs, and RS/CNTs. Peak deconvolution carried out by peak assignment to the secondary structures: 1650 cm^–1^ (random coil) and 1620 cm^–1^ (β-sheet) for amide I and 1540 cm^–1^ (random coil) for amide II. (b) Quantitative analysis of secondary structures in RS, RS/CNTs, and RS/f-CNTs films, respectively. (c) Stress/stretch curves for the RS, RS/CNTs, and RS/f-CNTs films. (d) Tensile strength and elongation at break of the prepared specimens calculated from tensile curves. DSC thermograms for the RS, RS/CNTs, and RS/f-CNTs films (e) before and (f) after UV irradiation, respectively. Dashed lines indicate the *T*_g_ values.

Structural changes of neat and composite RS films were investigated by FTIR spectroscopy. The spectra reported in Figure 2a clearly show the characteristic bands at 1600–1650 cm^–1^ of amide I and at 1520–1540 cm^–1^ for amide II.^44^ The amide I and II regions were deconvolved according to the peak positions of the conformations of the fibroin protein; 1650 cm^–1^ (random coil) and 1620 cm^–1^ (β-sheet) for amide I and 1540 cm^–1^ (random coil) for amide II were identified (Figure 2a).^45^

The ratios of the areas under the deconvolved curves of amide-I and amide-II peaks were used to quantify the percentage content of secondary structures as summarized in Figure 2b. The unmodified RS film shows broad amorphous amide-I and amide II peaks with high content of random coil and low content of β-sheet structures (Figure 2b). It is noteworthy that when unmodified CNTs are dispersed in the hosting RS matrix, we observed an increase of the crystalline fraction of β-sheets, even though the random coil structure is still the predominant one. On the contrary, the addition of f-CNTs leads to a very important increase (Figure 2b) of the β-sheet crystalline structure over the random coils, as evidenced by the intense band at 1622–1637 cm^–1^ (Figure 2a). These results are in agreement with a recent work indicating that β-sheet formation occurs via hydrogen bonding mediated by polar sidechains.^49^ The carboxyl-functionalized nanotubes form hydrogen bonds with the adjacent RS atoms; this consideration is important, especially in view of the following results, where we show that adhesive interactions with a substrate is enhanced via post-deposition induction of β-sheets.

The effect of the addition and content of f-CNTs on the tensile properties of the prepared specimens was also investigated. We obtained the best mechanical properties with 1 wt % of f-CNTs (Figure S1) with the RS/f-CNTs adhesive that has a modulus of 8 kPa (4 kPa for RS and 8 kPa for RS/CNTs, Figure 2c) and a stretch ratio at failure (defined as λ = *l*/*l*_0,_Figure 2d) more than 6 times the original length (*l*_0_). The addition of f-CNTs plasticizes the RS and thus increases the ultimate strain, leading to a tougher adhesive. Similar results have been recently obtained by Zhao et al.,^46^ who observed high elastic and stretchable silk scaffolds when CNTs are added to electrospun fibers of RS, and our results are confirmed by DSC investigations shown in Figure 2e, where with respect to neat RS, the addition of CNTs and f-CNTs has a plasticizing effect, leading to a decrease of *T*_g_ of about 20 °C.

Because the prepared materials were developed for a possible application in medicine, in order to evaluate the potential degradation due to the exposure to germicidal radiation, neat and composite RS films were exposed to UV-C light (20 min for each side of the sample was selected as irradiation time).^47^ By comparing DSC curves before and after UV-C irradiation (Figure 2e,f, respectively), no variations can be observed, and it can be assumed that no thermal modifications of RS due to the UV treatment occurred. Indeed, the reduction of the molecular weight of a polymer that may occur due to a sterilization process leads to the formation of low molecular weight fractions having different thermal properties with respect to the starting material.

**Figure 3:**
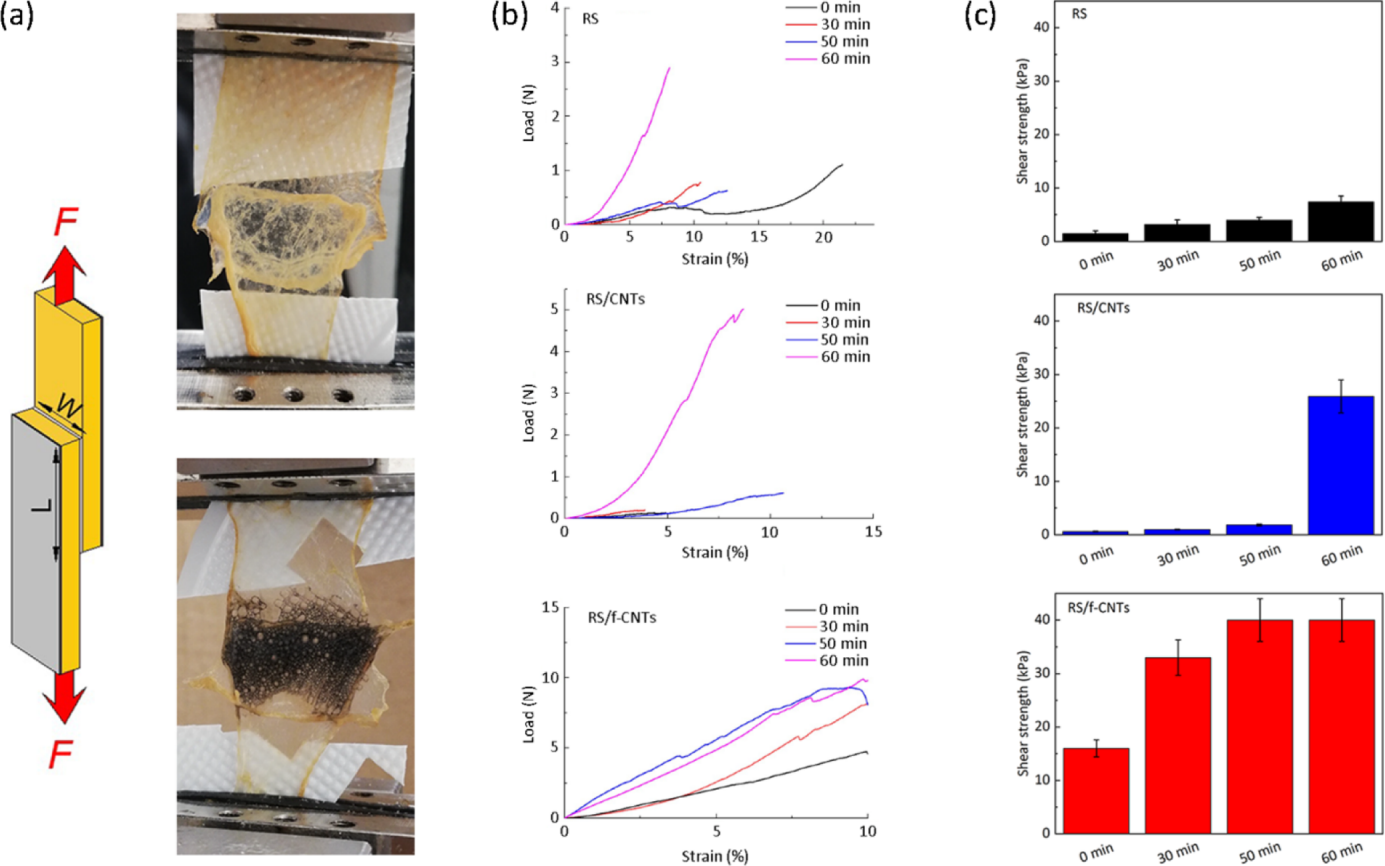
(a) Setup for measurement of shear strength (F, force; L, length; W, width). The photographs show RS (top) and RS/f-CNTs (bottom) inks adhered on porcine intestine. (b) Lap-shear tests and (c) shear strengths between porcine intestine of RS, RS/CNTs, and RS/f-CNTs adhesives as a function of the elapsed time from the deposition storing the samples at 37 °C and relative humidity of 65%.

The RS/f-CNTs have the consistency of a gel where the negatively charged carboxylic acid groups of the f-CNTs promote the water absorption from the wet tissue and simultaneously increase the ability to form hydrogen bonds with adjacent atoms of RS.^48,49^ In order to evaluate the effects of the carboxylic groups of CNTs on the silk fibroin sidechains on the adhesion to biological substrates, we measured the shear strength by lap-shear tests with RS and RS/f-CNTs adhesive films (Figure 3a,b). As a model substrate, we used porcine intestine portions that were overlapped and glued with neat and composite RS, respectively (Figure 3a). We choose wet porcine intestine due to its mechanical properties that are similar to those of the human skin.^50^ From the data reported in Figure 3b,c, the RS-based adhesive after 50 min shows better adhesive properties than that fabricated with RS/CNTs. Moreover, we observed a variation of the shear strength with elapsed time from the first test. In particular, the RS/f-CNTs samples were found to be more adhesive overall, reaching after 50 min a value of ≈40 kPa, which is higher than that recorded on RS (≈7 kPa) and RS/CNTs (≈25 kPa). These findings support the structural changes observed by FTIR analysis, confirming that hydrogen bonding is the driving mechanism that promotes the silk adhesion to the selected biological tissue.^49^ It should be noticed that the shear strength value recorded for RS/f-CNTs after 50 min is comparable to or even higher than that of commercial adhesives (Coseal ≈25 kPa, DuraSeal ≈12 kPa, Tisseel ≈12 kPa, and Tegaderm ≈50 kPa).^51−53^ The degradation of the adhesive properties has been also monitored for 12 days; the results reported in Figure S2 indicate that the adhesive properties last 8 days.

**Figure 4:**
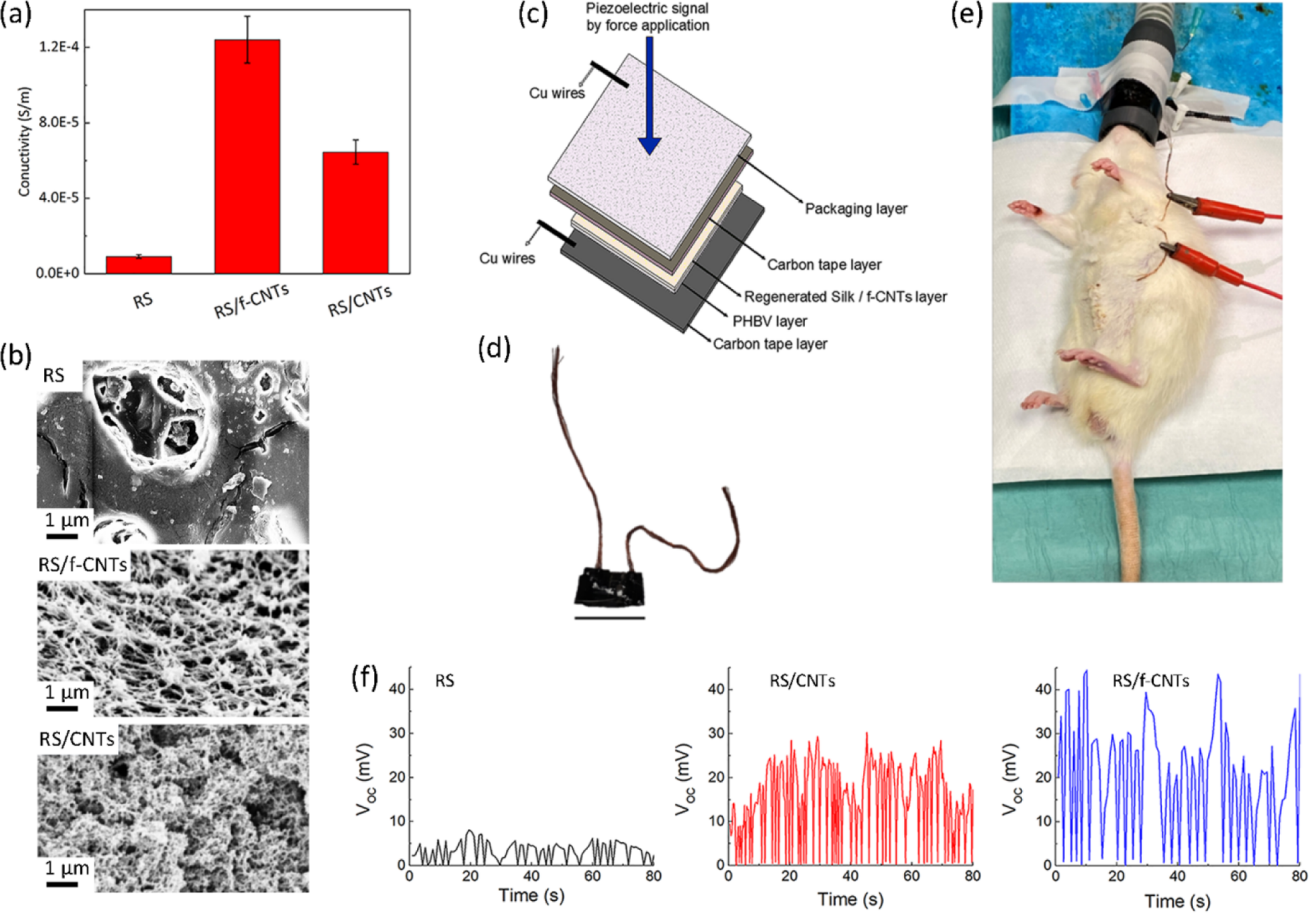
(a) Electrical conductivity of RS, RS/CNTs, and RS/f-CNTs films, respectively. (b) FESEM images of the surface morphologies of RS, RS/f-CNTs, and RS/CNTs films, respectively. (c) Schematic representation of the sensor layout and (d) Photograph of the fabricated piezoelectric device (scale bar indicates 1 cm). (e) Photograph illustrating the implanted sensor implanted sensor in a subcutaneous pocket of the rat abdomen. (f) Open-circuit voltage (*V*_OC_) data recorded by RS, RS/CNTs, and RS/f-CNTs films, showing the signal generated by the breathing of the rat under anesthesia.

To investigate the role played by the dispersion of CNTs on the electrical properties of the films, we measured the electrical conductivity, and the results are reported in Figures 4a and S3. Compared to the neat RS, the RS/f-CNT film exhibits an enhanced conductivity, despite the presence of functional groups that are known to increase the electrical resistivity.^54^ This result, if compared with the ones obtained on RS/CNTs, demonstrates the importance of the dispersion in the formation of a percolative path for the electron transport within RS (Figure 4a). Thus, this finding demonstrates the utility of employing re-dispersed RS solutions in water for the preparation of conductive silk-based inks.

The surface morphologies of RS, RS/f-CNTs, and RS/CNTs films were then investigated by field emission scanning electron microscopy (FESEM) analysis, and the results are reported in Figure 4b. When f-CNTs were added to the RS matrix, we observed a morphology made of filaments, where well-dispersed f-CNTs are wet by the fibroin protein without aggregation. On the contrary, the addition of unmodified CNTs in the hosting protein results in a clear morphological change. In particular, FESEM images in Figure 4b show aggregated structures with the loss of the filamentous morphology.

### 3.2 Piezoelectric Devices

In order to evaluate the possibility to fabricate smart patches, we adhere RS, RS/CNTs, and RS/f-CNTs on a PHBV film, a fully biobased and biodegradable polyester^55−57^ belonging to the poly(hydroxyalkanoate) (PHA) family. PHAs are biocompatible materials, which can be processed as a thermoplastic polymer sand and are frequently used in tissue engineering.^58,59^ In addition, PHAs can be used as a green alternative to acrylic and epoxy resins as substrates for circuits for the development of environmentally friendly electronic devices.^60^ We pave the utilization of RS composites on PHBV as a patch of different shapes to be applied on biological substrates with complex geometries.

As a proof of concept, we fabricated a piezoelectric force sensor by inserting the active bilayers (RS, RS/CNTs, and RS/f-CNTs on PHBV) between two carbon tape electrodes (Figure 4c,d). The fabricated self-powered sensor, once sterilized with Betadine, was inserted into a 2 × 2 cm^2^ subcutaneous pocket, which is made from the abdomen of a rat, as shown in Figure 4e.

Figure 4f shows the open-circuit voltage signals of RS, RS/CNTs, and RS/f-CNTs films, respectively. The output voltages reported in Figure 4e were obtained by the pressure of the diaphragmatic contraction due to the rat breathing under anesthesia. Considering the outputs generated from different samples, the device with the RS/f-CNTs film shows the largest difference between the maximum and minimum values. Furthermore, when we compare the difference between the press and release output signals of the samples reported in Figure 4f with the respective RS β-sheet content reported in Figure 2a, in general, we found that there is an increase of the piezoelectric performance with an increase of the β-sheet content.

**Figure 5:**
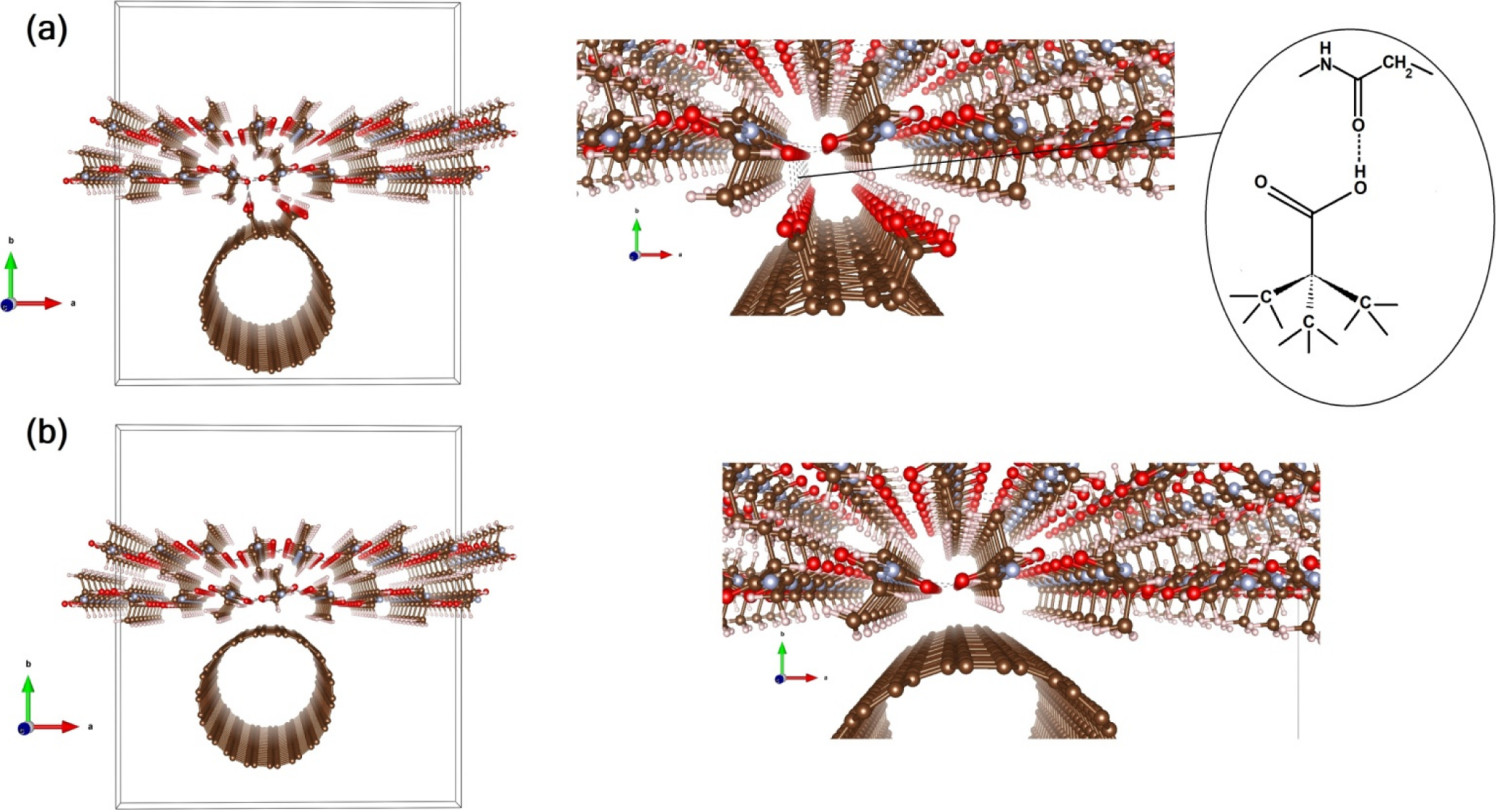
(a) Left: optimized geometry of the carboxyl-functionalized nanotube/fibroin interface. Right: zoom at the interfacial region (with a sketch of the stabilizing interaction between the functionalized nanotube and fibroin). (b) Same for the bare CNT/fibroin interface. The tetragonal solid line in both (a,b) describes the simulation box. (Red: O; Cyan: N; Brown: C; White: H atoms).

These findings (i.e., the dispersion grade and the electrical properties) may be explained by the optimization geometry of the two interfaces here investigated, that are, with and without tube carboxyl functionalization, as reported in Figure 5.

We calculated the adhesion energy (see eq 1) and found that for the bare CNT at the interface, it is exothermic by 0.44 eV. Interestingly, the functionalization of the tube with COOH moieties has the effect of doubling such *E*_adh_ up to 0.88 eV, testifying the paramount relevance of carboxyl terminations to thermodynamically favor the interface formation with silk fibroin. In addition to the adhesion energy, we have calculated the Bader charges for the two interfaces. The results reveal that once −COOH terminations^61^ are present, the interface charge transfer is nominally zero (Δ*Q* = ±0.03e), while the bare nanotube at the interface is accompanied by a more unbalanced, more “ionic”-like, distribution of the charges, with additional 0.15e extra charges that flow from the tube to the fibroin bilayer, further assessing the relevance of COOH terminations also in the charge transfer process at the interface.

### 3.3 3D-Printed Hollow Bilayer Cylindrical Structures

**Figure 6:**
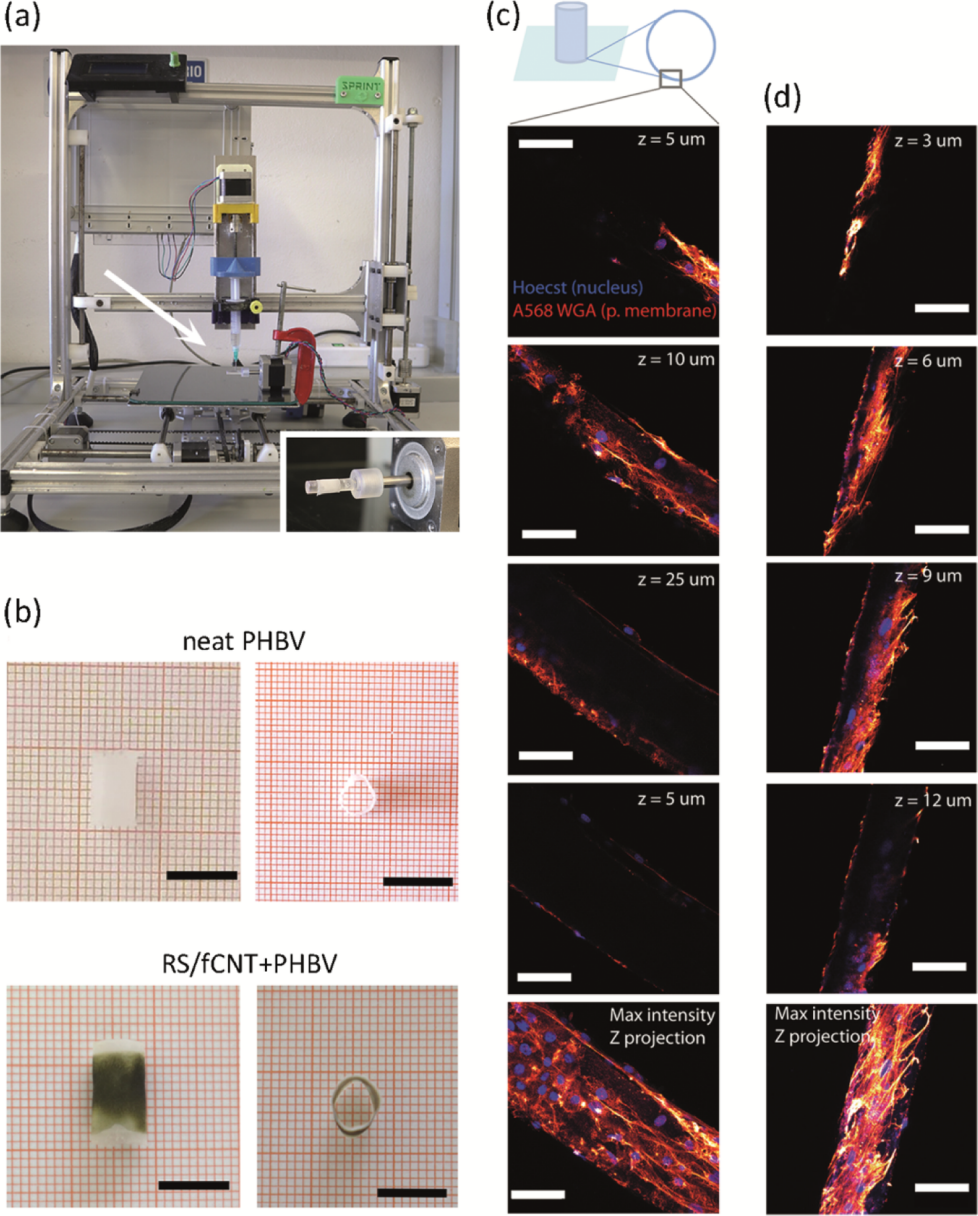
(a) Extrusion-based 3D printer, featuring a rotating spindle in the inset, that was used for the manufacturing of the hollow bilayer cylindrical structures. (b) Top and side view of the 3D-printed cylindrical structures, both monolayer (pure PHBV) and bilayer (RS/f-CNTS + PHBV). Scale bar = 1 cm. (c) Confocal microscopy images of human fibroblasts seeded on bilayer cylindrical structures after 21 days of incubation in standard conditions. Cells were labeled with the fluorescent Hoechst (blue channel) and WGA568 (red channel) dyes, which specifically target DNA and sialic acid, a component of the plasma membrane, respectively. The healthy condition of the cells clearly demonstrates that the RS/f-CNTs tube layer does not perturb cellular attachment and growth. Maximum intensity *Z* axis projection over 50 μm. (d) Confocal microscopy images of human fibroblasts seeded on PHBV tube after 21 days incubation in standard conditions. Cells were labeled with the fluorescent Hoechst (blue channel) and WGA568 (red channel) dyes. Scale bars in all panels indicate 25 μm.

A new material combination in 3D hollow bilayer cylindrical structures (Figures 6a and 6b) composed of an inner part, which is a cylindrical PHBV tube (1 cm length, 5 mm diameter) and an outer part that is a layer made of RS/f-CNTs with a thickness of approx. 50 μm,^14^ was investigated for tissue engineering applications. Human skin fibroblasts, one of the most widely studied cells, were used for preliminary assessment of biocompatibility. In Figure 6c, confocal microscopy images of fibroblasts attached on RS/f-CNTs + PHBV and pure PHBV structures are shown after 3 weeks of cell culture. The cells adhered on both structures and exhibited a healthy nuclear and overall shape and dimension. These findings indicate a favorable cell interaction with RS/f-CNTs and PHBV, and it is a prerequisite for designing 3D architectures for regenerative medicine experiments with these biomaterial inks. The biodegradation ratio (original weight loss) of RS and RS/f-CNTs after different times of degradation in PBS is reported in Figure S4 and indicates that the RS/f-CNT film degraded less than RS one, which is the degradation of about 11 wt % of the original weight lost after 12 days. The slowing down of the biodegradation can be ascribed to the high β-sheet content of RS when f-CNTs were added.

## 4 Conclusions

In this study, we have dispersed carboxyl-functionalized CNTs in the RS matrix through a solvent-free method. The RS/f-CNTs dispersion in the form of gel was used as liquid adhesive on a wet biological substrate. FTIR and mechanical analyses suggest that the RS adhesion is promoted by hydrogen bonding and that the presence of carboxy groups is fundamental to this aim. Thin solid films made of RS/f-CNTs exhibited an enhanced conductivity that makes possible the fabrication of a piezoelectric force sensor to monitor physiological forces. This sensor generates an open-circuit voltage upon applied force, and therefore in principle, it can be potentially envisioned as an adhesive on wet surface with energy-harvesting properties. The herein presented composite materials can be also exploited in the future as biomaterial inks for the preparation of biocompatible 3D-printed structures, where the presence of CNTs does not affect the surface bioactivity, suggesting that this new material combination could pave the way to applications in regenerative medicine.

## Supporting Information Available

Mechanical properties of RS/f-CNT composite films; adhesion properties of RS and RS composite films; electrical properties of RS composite films; and biodegradation of RS and RS composite films (PDF)
