# Supplementary material for: Carbon Nanotubes/Regenerated Silk Composite as a Three-Dimensional Printable Bio-Adhesive Ink with Self-Powering Properties
Source: ACS Appl Mater Interfaces. 2021 May 3;13(18):21007–17. doi: 10.1021/acsami.1c03288 (PMC8153539; doi:10.1021/acsami.1c03288)
Supplement: Supplementary file 1 — am1c03288_si_001.pdf [file am1c03288_si_001.pdf]

Supporting Information

Carbon Nanotubes/Regenerated Silk Composite as 3D-Printable Bio-Adhesive Ink with Self-Powering Properties

*Silvia Bittolo Bon [a,b], Irene Chiesa [c], Micaela Degli Esposti [b,d], Davide Morselli [b,d], Paola Fabbri [b,d], Carmelo De Maria [c], Antonino Morabito [e,f], Riccardo Coletta [e,g], Martino Calamai [h,i], Francesco Saverio Pavone [h,l], Rodolfo Tonin [m], Amelia Morrone [f,m], Giacomo Giorgi [n,o] and Luca Valentini [a,b,]**

[a] Dipartimento di Ingegneria Civile e Ambientale, Università degli Studi di Perugia, Strada di Pentima 4, 05100, Terni, Italy

[b] Italian Consortium for Science and Technology of Materials (INSTM), Via Giusti 9, 50121 Firenze, Italy

[c] Department of Ingegneria dell'Informazione and Research Center E. Piaggio, University of Pisa, Largo Lucio Lazzarino 1, 56122 Pisa, Italy

[d] Department of Civil Chemical, Environmental and Materials Engineering (DICAM), Università di Bologna, Via Terracini 28, Bologna 40131, Italy

[e] Department of Pediatric Surgery, Meyer Children's Hospital, Viale Pieraccini 24, 50139 Firenze, Italy

[f] Dipartimento Neuroscienze, Psicologia, Area del Farmaco e della Salute del Bambino NEUROFARBA, Università degli Studi di Firenze, Viale Pieraccini 6, 50121 Firenze, Italy

[g] School of Health and Society, University of Salford, Salford, United Kingdom

[h] European Laboratory for non-linear Spectroscopy (LENS), University of Florence, Sesto Fiorentino (FI), Italy

[i] National Institute of Optics -National Research Council (CNR-INO), Sesto Fiorentino (FI), Italy

[l] Department of Physics, University of Florence, Sesto Fiorentino (FI), Italy

[m] Molecular and Cell Biology Laboratory, Paediatric Neurology Unit and Laboratories, Neuroscience Department, Meyer Children's Hospital, Firenze, Italy

[n] Dipartimento di Ingegneria Civile e Ambientale (DICA), Università degli Studi di Perugia, Via G. Duranti 93, 06125, Perugia, Italy

* Corresponding Author: Luca Valentini (luca.valentini@unipg.it)

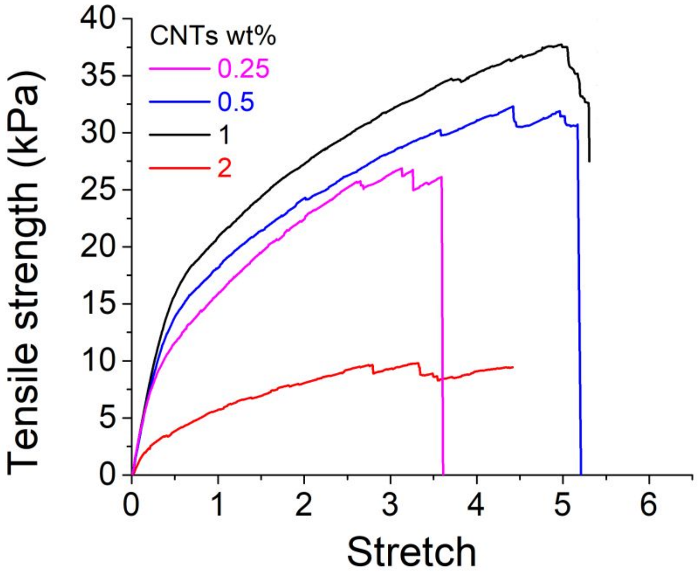

Figure S1. Stress/stretch curves for the RS/f-CNTs films with different f-CNTs content.

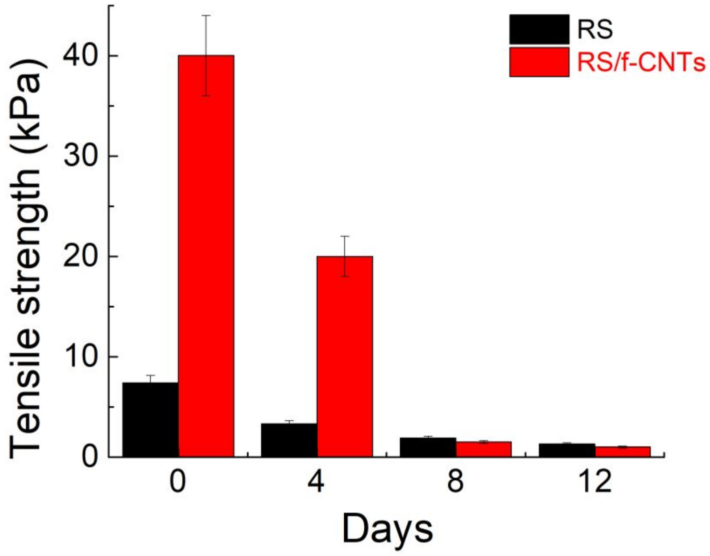

Figure S2. Shear strengths between porcine intestine and RS and RS/f-CNTs films as a function of the elapsed time from the preparation in environmental conditions.

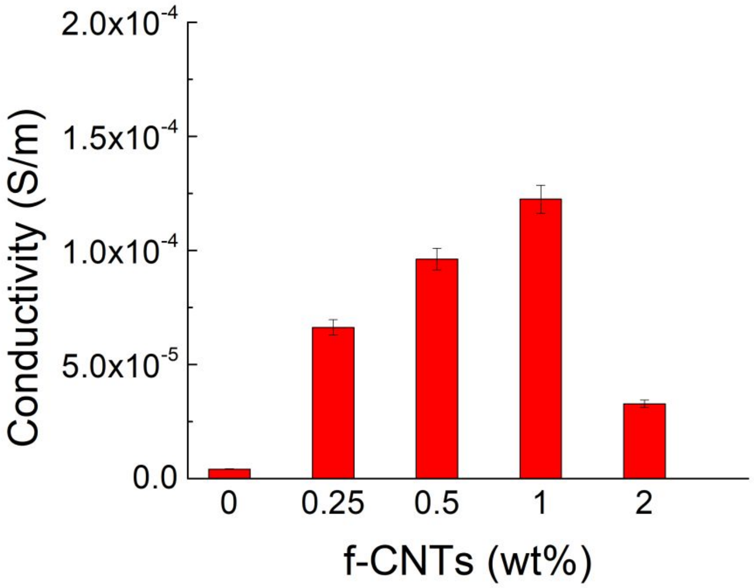

Figure S3. Electrical conductivity of RS/f-CNTs films as a function of different f-CNTs content.

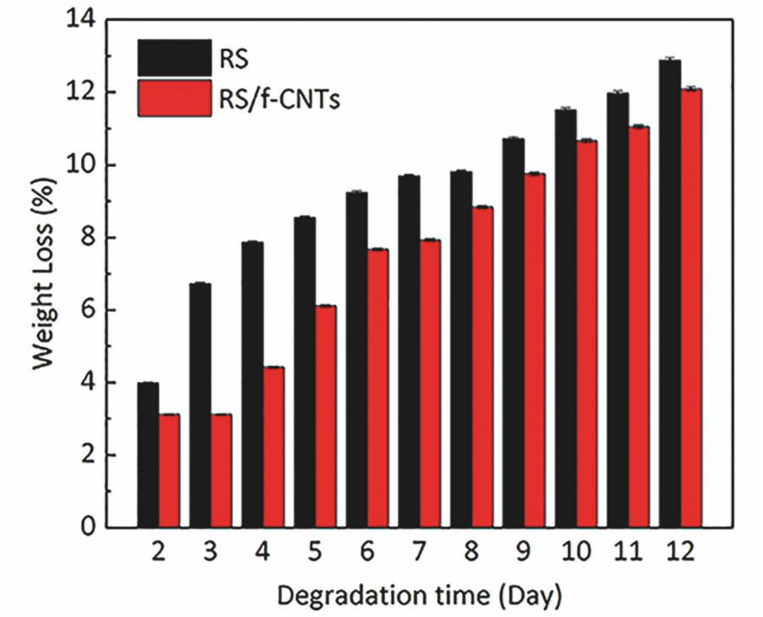

Figure S4. Weight loss as a function of the elapsed time of RS and RS/f-CNTs films in PBS, 1X Solution, pH 7.4, incubated at 37°C.
